# Improved chemistry restraints for crystallographic refinement by integrating the Amber force field into *Phenix*

**DOI:** 10.1107/S2059798319015134

**Published:** 2020-01-01

**Authors:** Nigel W. Moriarty, Pawel A. Janowski, Jason M. Swails, Hai Nguyen, Jane S. Richardson, David A. Case, Paul D. Adams

**Affiliations:** a Molecular Biosciences and Integrated Bioimaging, Lawrence Berkeley National Laboratory, Berkeley, CA 94720-8235, USA; b Department of Chemistry and Chemical Biology, Rutgers University, Piscataway, NJ 08854, USA; c Department of Biochemistry, Duke University, Durham, NC 27710, USA; d Department of Bioengineering, University of California at Berkeley, Berkeley, CA 94720, USA

**Keywords:** *Amber* refinement target, hydrogen-bond quality, *Amber* in *Phenix*, C^β^ deviations, peptide orientations

## Abstract

The refinement of biomolecular crystallographic models relies on geometric restraints to help to address the paucity of experimental data typical in these experiments. Limitations in these restraints can degrade the quality of the resulting atomic models. Here, an integration of the full all-atom Amber molecular-dynamics force field into *Phenix* crystallographic refinement is presented, which enables more complete modeling of biomolecular chemistry. The advantages of the force field include a carefully derived set of torsion-angle potentials, an extensive and flexible set of atom types, Lennard–Jones treatment of nonbonded interactions and a full treatment of crystalline electrostatics. The new combined method was tested against conventional geometry restraints for over 22 000 protein structures. Structures refined with the new method show substantially improved model quality. On average, Ramachandran and rotamer scores are somewhat better, clashscores and *MolProbity* scores are significantly improved, and the modeling of electrostatics leads to structures that exhibit more, and more correct, hydrogen bonds than those refined using traditional geometry restraints. In general it is found that model improvements are greatest at lower resolutions, prompting plans to add the *Amber* target function to real-space refinement for use in electron cryo-microscopy. This work opens the door to the future development of more advanced applications such as *Amber*-based ensemble refinement, quantum-mechanical representation of active sites and improved geometric restraints for simulated annealing.

## Abstract 2

The full Amber force field has been integrated into *Phenix* as an alternative refinement target. With a slight loss in speed, it achieves improved stereochemistry, fewer steric clashes and better hydrogen bonds.

## 1. Introduction   

Accurate structural knowledge lies at the heart of our understanding of the biomolecular function and interactions of proteins and nucleic acids. With close to 90% of the structures in the Protein Data Bank (Berman *et al.*, 2000 ▸) solved via X-ray diffraction methods, crystallography is currently the pre-eminent method for determining biomolecular structure. Crystal structure refinement is a computational technique that plays a key role in post-experiment data interpretation. The refinement of atomic coordinates entails solving an optimization problem to minimize the residual difference between the experimental and model structure-factor amplitudes (Jack & Levitt, 1978 ▸; Agarwal, 1978 ▸; Murshudov *et al.*, 1997 ▸). However, owing to inherent experimental limitations and a typically low data-to-parameter ratio, the employment of additional restraints, commonly referred to as geometry or steric restraints, is key to successful structural refinement (Waser, 1963 ▸). These restraints, which can be thought of as a prior in the Bayesian sense, provide additional observations in the optimization target and reduce the danger of overfitting. Their use leads to higher quality, more chemically accurate models.

Most current refinement programs (Afonine *et al.*, 2012 ▸; Murshudov *et al.*, 2011 ▸; Sheldrick, 2015 ▸; Bricogne *et al.*, 2011 ▸) employ a set of covalent geometry restraints first proposed by Engh and Huber in 1991 and later augmented and improved in 2001 (Engh & Huber, 1991 ▸, 2001 ▸). This set of restraints is based on a survey of accurate high-resolution small-molecule crystal structures from the Cambridge Structural Database (Groom *et al.*, 2016 ▸) and includes restraints on interatomic bond lengths, bond angles and ω torsion angles. In addition, parameters are added to enforce proper chirality and planarity, multiple-minimum targets for backbone and side-chain torsion angles, and repulsive terms to prevent steric overlap between atoms. Those terms are defined from small-molecule and high-resolution macromolecular crystal structure data and from interaction-specified van der Waals radii. They are very similar, but not identical, between refinement programs.

The Engh and Huber restraints function reasonably well, while the additional terms have been gradually improved, but a number of limitations have been identified over the years. Some of these limitations include a lack of adjustability to differences in local conformation, protonation and hydrogen bonding and to their changes during refinement, incomplete or inaccurate atom types and parameters for ligands, carbo­hydrates and covalent modifications, the use of only repulsive and not attractive steric terms, the omission of explicit H atoms and their interactions, misleading targets resulting from experimental averaging artifacts, inaccurate dihedral restraints, and a lack of awareness of electrostatic and quantum dispersive interactions, with a consequent lack of accounting for hydrogen-bonding cooperativity (Priestle, 2003 ▸; Touw & Vriend, 2010 ▸; Davis *et al.*, 2003 ▸; Moriarty *et al.*, 2014 ▸; Tronrud *et al.*, 2010 ▸).

*Phenix* (Liebschner *et al.*, 2019 ▸) includes a built-in system for defining ligand parameters (Moriarty *et al.*, 2009 ▸) that by default restrains the explicit H atoms at electron-cloud center positions for X-ray crystallography and optionally at nuclear positions for neutron crystallography (Williams, Headd *et al.*, 2018 ▸). The addition of the Conformation Dependent Library (CDL; Moriarty *et al.*, 2014 ▸), which makes backbone bond lengths and angles dependent on φ, ψ values, has improved the models obtained from refinement at all resolutions, and thus is the default in *Phenix* refinement (Moriarty *et al.*, 2016 ▸). Similarly, *Phenix* uses ribose-pucker- and base-type-dependent torsional restraints for RNA (Jain *et al.*, 2015 ▸). For bond lengths and angles, protein side chains continue to use standard Engh and Huber restraints, while RNA/DNA use early values (Parkinson *et al.*, 1996 ▸) with a few modifications. This use of combined restraints is here designated CDL/E&H.

An alternative approach is the use of geometry restraints based on the all-atom force fields used for molecular-dynamics studies. This is not a novel idea. In fact, some of the earliest implementations of refinement programs employed molecular-mechanics force fields (Jack & Levitt, 1978 ▸; Brünger *et al.*, 1987 ▸, 1989 ▸). However, at the time, restraints derived from the coordinates of ideal fragments (Tronrud *et al.*, 1987 ▸; Hendrickson & Konnert, 1980 ▸) were found to provide better refinement results. The insufficiency of molecular-mechanics-based restraints was mainly attributed to two factors: an inaccurate representation of chemical space because of too few atom types, and biases in conformational sampling resulting from unshielded electrostatic interactions. Subsequently, however, the methods of molecular dynamics and the corresponding force fields have seen significant development and improvement. Current force fields contain more atom types and are easily adjustable as needed. They are typically parameterized against accurate quantum-mechanical calculations, which was not feasible just a few years ago, as well as using more representative experimental results. Significant methodological advances, such as the development of the particle mesh Ewald method (York *et al.*, 1993 ▸; Darden *et al.*, 1993 ▸) for the accurate calculation of crystalline electrostatics and improved temperature- and pressure-control algorithms, have greatly increased accuracy. Modern force fields have been shown to agree well with experimental data (Zagrovic *et al.*, 2008 ▸; van Gunsteren *et al.*, 2008 ▸; Showalter & Brüschweiler, 2007 ▸; Grindon *et al.*, 2004 ▸; Bowman *et al.*, 2011 ▸), including crystal diffraction data (Cerutti *et al.*, 2008 ▸, 2009 ▸; Janowski *et al.*, 2013 ▸, 2015 ▸; Liu *et al.*, 2015 ▸).

We have made it possible to use the Amber molecular-mechanics force field as an alternative source of geometry restraints to those from CDL/E&H. Here, we present an integration of the *Phenix* software package for crystallo­graphic refinement, *phenix.refine* (Afonine *et al.*, 2012 ▸), and the *Amber* software package (Case *et al.*, 2018 ▸) for molecular dynamics. We present results of paired refinements for 22 544 structures and compare *Amber* with traditional refinement in terms of model quality, chemical accuracy and agreement with experimental data, studied both for overall statistics and for representative individual examples. We also describe the implementation and discuss future directions.

## 2. Methods   

### 2.1. Code preparation   

The integration of the *Amber* code into *phenix.refine* uses a thin client. *Amber* provides a Python API to its *sander* module, so that a simple ‘import sander’ Python command allows *Phenix* to obtain Amber energies and forces through a method call. At each step of coordinate refinement, *Phenix* expands the asymmetric unit coordinates to a full unit cell (as required by *sander*), combines energy gradients returned from *Amber* (in place of those from its internal geometric restraint routines) with gradients from the X-ray target function, and uses these forces to update the coordinates. Alternate conformers can take advantage of the ‘locally enchanced sampling’ (LES) facility in *sander*: atoms in single-conformer regions interact with multiple-copy regions via the average energy of interaction, while different copies of the same group do not interact among themselves (Roitberg & Elber, 1991 ▸; Simmerling *et al.*, 1998 ▸).

The *Amber* files required are created by a preliminary *AmberPrep* program that takes a PDB file as input. It creates both a parameter-topology (prmtop) file used by *Amber* and a new PDB file containing a complete set of atoms (including hydrogens and any missing atoms) needed to perform force-field calculations. If requested, alternate conformers present in the input PDB file can be translated into *sander* LES format. For most situations, *AmberPrep* does not require the user to have any experience with *Amber* or with molecular mechanics; less-common situations (described in the supporting information) require some familiarity with *Amber*. All of the code required for both the *AmberPrep* and *phenix.refine* steps is included in the current major release, v.1.16-3549, and subsequent nightly builds of *Phenix*.

### 2.2. Structure selection and overall refinement protocol   

To compare refinements using *Amber* against traditional refinements with CDL/E&H restraints, structures were selected from the Protein Data Bank (PDB; Burley *et al.*, 2019 ▸) using the following criteria. Entries must have untwinned experimental data available that are at least 90% complete. For each entry, *R*_free_ was limited to a maximum of 35%, *R*_work_ to 30% and *R*_Δ_ (*R*_free_ − *R*_work_) to a minimum of 1.5%. The lowest resolution was set at 3.65 Å. Entries containing nucleic acids were excluded.

**Figure 1:**
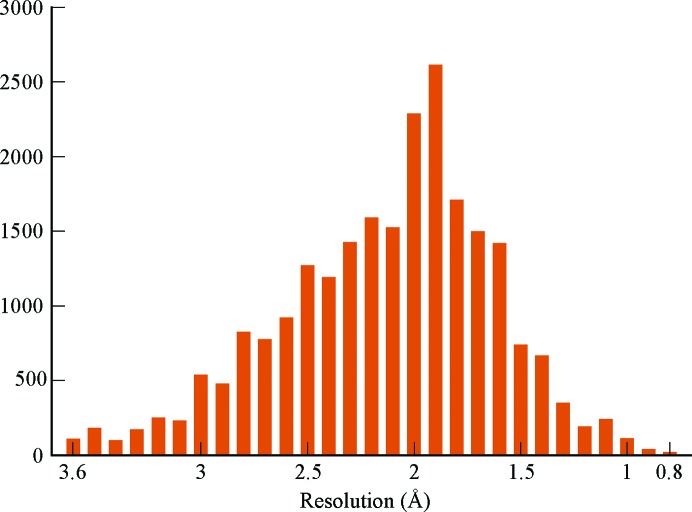
Distribution of refined structures across resolution bins.

Coordinate and experimental data files were obtained directly from the PDB and inputs were prepared via the automated *AmberPrep* program (see Section 2.1). Entries containing complex ligands were included if the file-preparation program *AmberPrep* was able to automatically generate and include the ligand geometry data; this generally excludes ligands containing covalent connections to the protein or with metal atoms. Details of the internals of *AmberPrep* will be described elsewhere. Resolution bins (set at 0.1 Å) with less than ten refinement pairs were eliminated to reduce the noise caused by limited statistics. Complete graphs are included in the supporting information. The resulting 22 000+ structures had experimental data resolutions between 0.8 and 3.6 Å, with most of the structures in the 1.2–3.0 Å range (see Fig. 1 ▸).

Each model was then subjected to ten macrocyles of refinement using the default strategy in *phenix.refine* for reciprocal-space coordinate refinement, with the exception that real-space refinement was turned off. By default, the first macrocycle uses a least-squares target function and the rest use maximum likelihood. Other options applied to both CDL/E&H and *Amber* refinements included optimization of the weight between the experimental data and the geometry restraints. This protocol was performed in parallel, once using CDL/E&H and once using *Amber* geometry restraints. In addition, C^β^ pseudo-torsion restraints were not included in the restraints model. Explicit parameter settings are included in the supporting information. Only one copy of each alternate conformation was considered initially (*i.e.* alternative location A). The final files are available by contacting the corresponding author.

The quality of the resulting models was assessed numerically using *MolProbity* (Williams, Headd *et al.*, 2018 ▸) available in *Phenix* (Adams *et al.*, 2010 ▸), by *cpptraj* (Roe & Cheatham, 2013 ▸) available in *AmberTools* (Case *et al.*, 2018 ▸) and by visual inspection with electron-density and validation markup in *KiNG* (Chen *et al.*, 2009 ▸). All-atom dots for Fig. 10 were counted in *Mage* (Richardson & Richardson, 2001 ▸) and Figs. 5–9 were made in *KiNG*. To avoid typographical ambiguity, PDB codes are given here in lower case for all letters except L (for example 1nLs; Moriarty, 2015 ▸).

### 2.3. Weight-factor details   

The target function optimized in *phenix.refine* reciprocal-space atomic coordinate refinement is of the general form
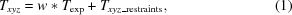
where all of the terms are functions of the atomic coordinates, *T_xyz_* is the target residual to be minimized, *T*_exp_ is a residual between the observed and model structure factors and quantifies agreement with experimental data, *T*_*xyz*_restraints_ is the residual of agreement with the geometry restraints and *w* is a scale factor that modulates the relative weight between the experimental and the geometry restraint terms. In traditional refinement *T*_*xyz*_restraints_ is calculated using the set of CDL/E&H restraints,
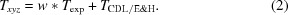



To implement *Phenix*–*Amber* we substitute this term with the potential energy calculated using the Amber force field,
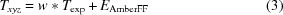
where the *Amber* term is intentionally represented now by an *E* to emphasize that we directly incorporate the full potential energy function calculated in *Amber* using the ff14SB force field (Maier *et al.*, 2015 ▸).

In a standard default *Phenix* refinement, the weight *w* is a combination of a value based on the ratio of gradient norms (Brünger *et al.*, 1989 ▸; Adams *et al.*, 1997 ▸) and a scaling factor that defaults to 0.5. This initial weight can be optimized using a procedure described previously (Afonine *et al.*, 2011 ▸). This procedure uses the results of ten refinements with a selection of weights, considering the bond and angle r.m.s.d., the *R* factors and validation statistics to determine the best weight for the specific refinement at each of the ten macrocycles. The same procedure was used to estimate an optimal weight for the *Phenix*–*Amber* refinements. (If faster fixed-weight refinements are desired, we have found that a scaling factor of 0.2, rather than 0.5, scales the *Amber* gradients to be close to those from the CDL/E&H restraints, allowing the simpler, default, weighting scheme in *phenix.refine* to be used.)

## 3. Results   

### 3.1. Full-data-set score comparisons   

**Figure 2:**
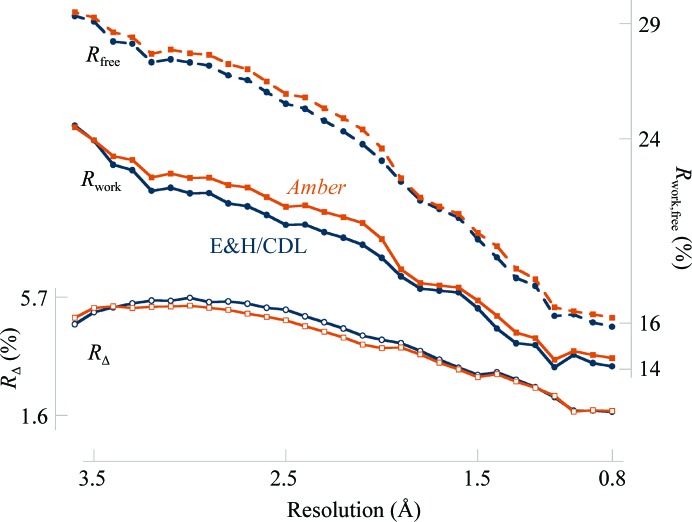
*R* factors of optimized weight refinements and *R*_free_ − *R*_work_ (*R*_Δ_) versus resolution (values averaged in each resolution bin). Vertical axes are in % with the *R*_Δ_ axis on the left. E&H/CDL values are plotted in dark blue and those for *Amber* in burnt orange.

**Figure 3:**
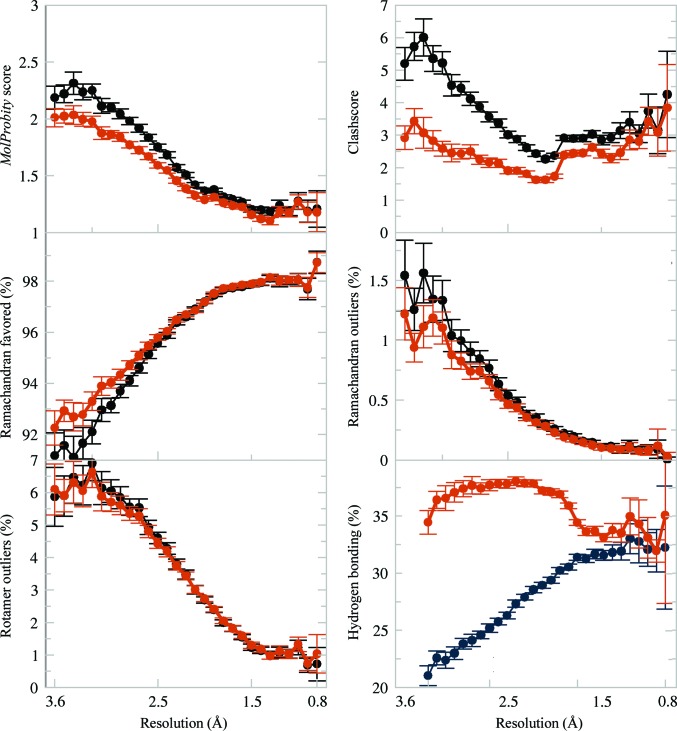
Comparison plots of model-quality measures versus resolution for *Amber* (burnt orange) versus CDL/E&H (dark blue) refinements with error bars depicting the 95% confidence level of the standard error of the mean. The *MolProbity* score is a combination of all-atom clashscore and Ramachandran favored and rotamer outliers, weighted to approximate the expected score at the resolution of the structure. The hydrogen-bond fraction is calculated using *cpptraj* per 1000 atoms in the model. For all six plots, *Amber* (burnt orange) differs in the better direction.

On average, the *Phenix*–*Amber* combination produced slightly higher *R*_work_ and *R*_free_ values (Fig. 2 ▸) but higher quality models (Fig. 3 ▸). The increase in *R* factors is most pronounced in the 1.8–2.8 Å range. This is a result of the weight-optimization procedure having different limits for optimal weight in this resolution range. The increase was less for *R*_free_ than *R*_work_, such that *R*_Δ_ is less for refinements using *Amber* gradients. The uncertainty in the *R*_free_ for 95% of refinements calculated using equation (13) of Tickle *et al.* (2000 ▸) is less than 0.032. At 2 Å resolution, this equates to an uncertainty of 0.7%, which is approximately the same as the difference in the average *R*_free_ values of 23.0% and 23.6% for *Phenix* and *Phenix*–*Amber*, respectively.

The *Phenix*–*Amber* refinements exhibited improved (lower) *MolProbity* scores and contained fewer clashes between atoms. Plots show the mean of the values in the 0.1 Å resolution bin as well as the 95% confidence level of the standard error of the mean (SEM). The *MolProbity* clashscores are particularly striking: for refinement using CDL/E&H restraints the clashscores steadily increase as resolution worsens, often resulting in very high numbers of steric clashes. On the other hand, the mean clashscore with *Amber* restraints appears to be nearly independent of resolution and remains consistent at about 2.5 clashes per 1000 atoms across all resolution bins. The SEM range is non-overlapping at worse than 1 Å, indicating that the Amber force field is producing better geometries at mid to low resolution. There are more favored Ramachandran points (backbone φ, ψ) and fewer Ramachandran outliers for the *Phenix*–*Amber* refinements. This difference is most marked for resolutions worse than 2 Å. *Phenix*–*Amber* refinement also improves (lowers) the number of rotamer outliers but does not differentiate via the SEM, and increases the proportion of hydrogen bonds. While the rotamer outlier results remain similar, the hydrogen-bonding results have a large difference at worse than 2 Å, resulting in nearly double the bonds near 3 Å. Common to all the plots is a change near 2 Å, where the weight-optimization procedure common to both CDL/E&H and *Amber* refinement loosens the weight on geometry restraints somewhat to allow more deviations at resolutions where the data are capable of unambiguously showing them. Bond and angle r.m.s.d. comparisons are less pertinent as the force fields do not have ideal values for parameterizations and comparing the *Phenix*–*Amber* bonds and angles with the CDL/E&H values is not a universal metric. The curious can see the plots in Supplementary Fig. S1. Overall, the improvement with *Amber* is substantial in the lower resolution refinements.

**Figure 4:**
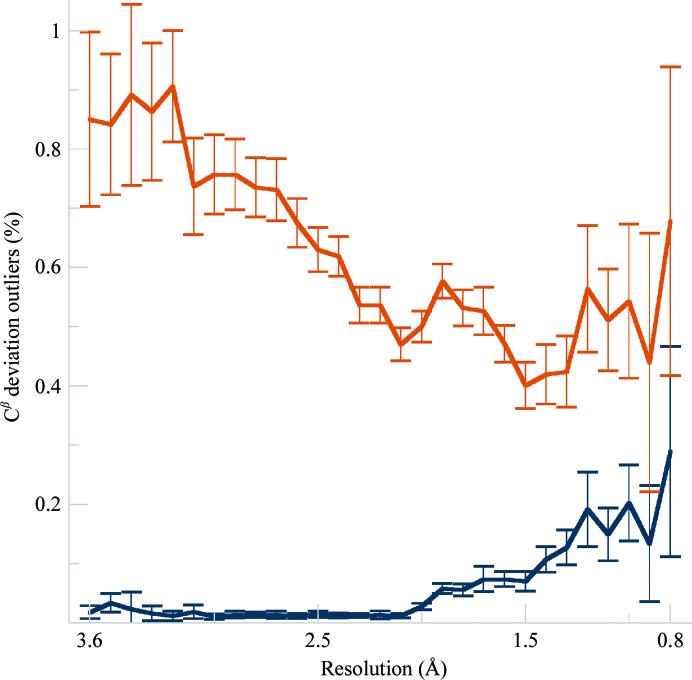
Fraction of C^β^ deviations (in %) per C^β^ atom as a function of resolution for the CDL/E&H (dark blue) and *Amber* (burnt orange) refinements. Values are averaged in each bin of resolution, with the error bars showing the 95% confidence level of the standard error of the mean.

One validation metric that is worse for *Phenix*–*Amber* refinements is the number of outliers for C^β^ positions. Both the mean and the SEM show clear differentiation. The C^β^ deviation (Cβd) is the distance between the modeled C^β^ and an ideal C^β^, which is a combined measure of distortion in the tetrahedron around the C^α^ atom. The ideal position is calculated by averaging the N—C—C^α^—C^β^ and C—N—C^α^—C^β^ improper dihedrals and correcting the bond length, which allows for the effect of a non-ideal τ angle (Lovell *et al.*, 2003 ▸). With traditional E&H restraints the Cβd is quite robustly sensitive to incompatibility between how the backbone and side-chain conformations have been modeled. For CDL/E&H refinements, however, the percentage of Cβd outliers (>0.25 Å) is negligible for low and mid resolutions, only increasing to 0.2% at higher resolutions (see Fig. 4 ▸). This is in line with CDL/E&H providing tight geometrical restraints out to C^β^ at most resolutions, but loosened somewhat at better than 2 Å resolution, where there is sufficient experimental information to move an angle away from ideal. Note that explicit C^β^ restraints were turned off for all *Phenix* refinements and that the Amber force field does not have an explicit C^β^ term; however, if all angles around the C^α^ atom are kept ideal then the C^β^ position will also be ideal even if it is incorrectly positioned in the structure. The following section analyses specific local examples where output structures show differences for either the positive or the negative trends seen in the overall comparisons, in order to understand their nature, causes and meaning across resolution ranges.

### 3.2. Examination of individual examples   

As noted above, in comparison with the CDL/E&H restraint refinements, the *Phenix*–*Amber* refinements have much higher percentages of C^β^ deviation outliers, increasing at the low-resolution end to more than 1% of C^β^ atoms. *Amber* refinement also has more bond-length and angle outliers. The following examines a sample of cases at high, mid and lower resolutions to understand the starting-model characteristics and refinement behavior that produce these differences.

#### 3.2.1. High resolution: waters, alternates, Cβd outliers and atoms in the wrong peak   

In the high-resolution range (better than 1.7 Å), it appears that the commonest problems that are not easily correctable by refinement are caused either by modeling the wrong atom into a density peak or by incorrect modeling, labeling or truncation of alternate conformations. Such problems are usually flagged in validation either by all-atom clashes, by C^β^ deviations and sometimes by bad bond lengths and angles. (For the high-resolution examples described here, we used the LES procedure outlined above to model alternative conformers in the *Phenix*–*Amber* refinements.)

**Figure 5:**
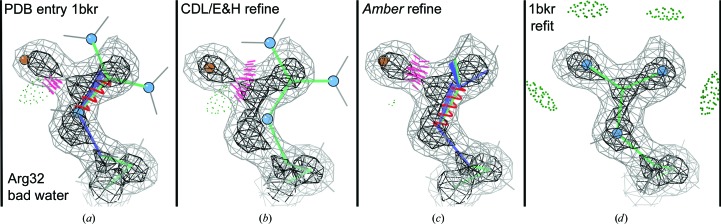
Differing responses of CDL/E&H versus *Amber* refinement to the misfitting of a water into what should be a side-chain N atom in an arginine residue. Neither result here is acceptable, but if the incorrect water is deleted (*d*) then both methods do a very good job of moving the guanidinium correctly back into its density. *MolProbity* markup for Figs. 5–10: clusters of hot pink spikes represent clashes, pillows of green dots represent hydrogen bonds, red or blue springs or fans represent larger or smaller bond-length or angle outliers, magenta spheres represent C^β^ deviations, gold side chains represent rotamer outliers, green C^α^–C^α^ lines represent Ramachandran outliers and magenta lines along the CO–CO dihedral represent *CaBLAM* outliers. Relevantly moving O or N atoms are emphasized with red or blue spheres.

Fig. 5 ▸(*a*) shows a case in which a water molecule had been modeled in an electron-density peak that should really be an N atom of an arginine guanidinium. CDL/E&H refinement (Fig. 5 ▸ *b*) corrected the bad geometry at the cost of moving the guanidinium even further out of density; *Amber* refinement changed the orientation of the guanidinium but made no overall improvement (Fig. 5 ▸ *c*); all three versions have a bad clash. If the water were deleted then either refinement method would undoubtedly do an excellent job (Fig. 5 ▸ *d*). This type of problem is absent at low resolution, where waters are not modeled, but occurs quite often at both high and mid resolution for other branched side chains, for Ile C^δ^ (for example, Ile195 in PDB entry 3js8) and even occasionally for Trp (for example, TrpB170 in PDB entry 1qw9).

**Figure 6:**
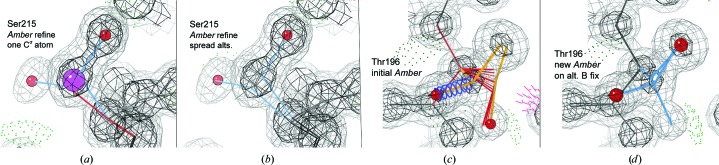
At high resolution, C^β^ deviation outliers are most often due to problems with alternate conformations. (*a*) *Amber* refinement using the original Ser215 alternates in PDB entry 1nLs, which have widely separated positions for C^β^ but only a single C^α^ atom. (*b*) *Amber* refinement after the definition of alternates has been spread to include the C^α^ and both adjoining peptides. (*c*) *Amber* refinement of the original Thr196 of PDB entry 1nLs, where alternate B had been fitted backwards; there is bad covalent geometry and a huge Cβd of 0.88 Å (sphere not shown). (*d*) The good *Amber* result after alternate B was refitted in the correct rotamer so that all atoms match the density.

C^β^ deviation outliers (≥0.25 Å) are often produced by side-chain alternates with quite different C^β^ positions but with no associated alternates defined along the backbone. Since the tetrahedron around C^α^ should be nearly ideal, this treatment almost guarantees bad geometry. The rather simple solution, implemented in *Phenix*, is to define alternates for all atoms until the *i* + 1 and *i* − 1 C^α^ atoms, as in the ‘backrub’ motion (Davis *et al.*, 2006 ▸). PDB entries 1dy5, 1gwe and 1nLs each have a number of such cases. Figs. 6 ▸(*a*) and 6 ▸(*b*) show Ser215 in PDB entry 1nLs, initially with an outlier Cβd, a distance of 0.49 Å between the two C^β^ atoms and a single C^α^ atom. CDL/E&H refinement pulls the C^β^ atoms to be only 0.23 Å apart, avoiding a Cβd with only slightly worse fit to the density; *Amber* reduces the Cβd only slightly, but it does keep this flag of an underlying problem. When alternates are defined for the backbone peptides, both systems improve.

Worse cases occur where one or both alternates have been fitted incorrectly as well as not being expanded along the backbone appropriately. Fig. 6 ▸(*c*) shows Thr196, with a huge Cβd of 0.88 Å (sphere not shown) and very poor geometry because alternate B was fitted incorrectly (just as a shift of alternate A rather than as a new rotamer). This time even CDL/E&H refinement produces a Cβd outlier, but smaller than that for *Amber*. Fig. 6 ▸(*d*) shows the excellent *Amber* result after the misfitting of alternate B was approximately corrected.

#### 3.2.2. Mid resolution: backward side chains and rare conformations   

**Figure 7:**
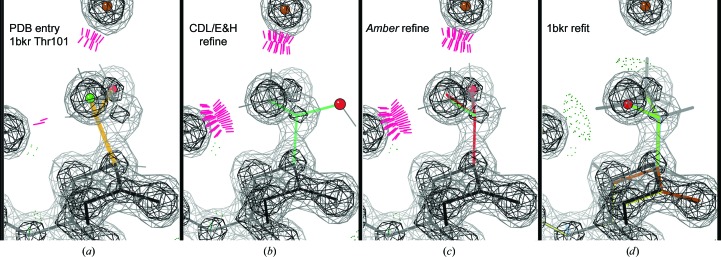
Unacceptable ways of getting rid of a C^β^ deviation without fixing the actual problem. (*a*) Thr101 in PDB entry 1bkr as deposited, with a huge Cβd of 0.63 Å (not shown as a sphere because it obscures the side chain), clashes, a rotamer outlier, the heavier O^γ^ branch in the lower electron-density peak and the C^β^ out of density, all of which are caused by modeling the side chain χ_1_ 180° backwards. (*b*) CDL/E&H makes the geometry perfect but places the O^γ^ far out of density. (*c*) *Amber* places all three side-chain atoms into peaks by making the chirality at C^β^ incorrect. (*d*) A refit in the correct rotamer replaces clashes with hydrogen bonds, has no outliers and puts each atom into its correct density peak.

An even commoner case at both high and mid resolutions where the wrong atom is fitted into a density peak is a backward-fitted C^β^-branched residue, which is well illustrated by a very clear Thr example in PDB entry 1bkr at 1.1 Å resolution (Fig. 7 ▸ *a*). Thr101 is a rotamer outlier (gold) on a regular α-helix with a Cβd of 0.63 Å. The deposited Thr101 also has a bond-angle deviation of 13.5σ, clashes at the C^γ^ methyl, its C^β^ is out of density, O^γ^ is in the lower peak and C^γ^ is in the higher peak. It is shown in Fig. 7 ▸ with 1.6σ and 4σ 2*mF*_o_ − *DF*_c_ contours (but without C^β^ deviation and angle markups for clarity). This mistake was not obvious because anisotropic *B* factors were used too early in the modeling, resulting in the Thr C^β^ being refined to a 6:1 aniso-axis ratio that covered both the modeled atom and the real position. The figures show the density as calculated with isotropic *B* factors.

Given this difficult problem for automated refinement, each of the two target functions reacts very differently. Both refinements still have the C^γ^ methyl clashing with a helix backbone CO in good density, which is very diagnostic of a problem with the C^γ^ atom. It is indeed the wrong atom to have in this peak, as is also shown by the relative peak heights. The CDL/E&H refinement (Fig. 7 ▸ *b*) achieves tight geometry and a good rotamer, moving the C^β^ atom into its correct density peak, but pays the price for not correcting the underlying problem by swinging the O^γ^ atom out of density. The *Amber* refinement (Fig. 7 ▸ *c*) achieves an atom in each of the three side-chain density peaks, but pays the price for not correcting the underlying problem by having the wrong chirality at the C^β^ atom. It still also has bond-angle outliers, which may be a sign of unconverged refinement.

The original PDB entry, the CDL/E&H refinement and the *Amber* refinement structures for Thr101 are all very badly wrong, but each in an entirely different way. The deposited model, PDB entry 1bkr, looks very poor by traditional model validation, but has a misleadingly good density correlation given the extremely anisotropic C^β^ *B* factor. The CDL/E&H output looks extremely good on traditional validation except for the clashes and would show a lowered but still reasonable density correlation; however, it is the most obviously wrong upon manual inspection. The *Amber* output has clashes and currently has modest bond-angle outliers, but it fits the density very closely, making it difficult to identify as incorrect by visual inspection. The problem could be recognized automatically by a simple chirality check. As shown in Fig. 7 ▸(*d*), Thr101 was rebuilt quickly in *KiNG* with the **p** rotamer and a small backrub motion. Either *Phenix*–CDL/E&H or *Phenix*–*Amber* refinement would do a very good job from such a rough refit with the correct atoms near the right places.

At mid resolution, there are also other rotamers and backbone conformations fitted into the wrong local minimum, and thus difficult to correct by minimization refinement methods, but not always flagged by C^β^ deviations or other outliers. Some of these, such as *cis*-nonproline peptides (Williams, Videau *et al.*, 2018 ▸) or very rare rotamers (Hintze *et al.*, 2016 ▸), can be avoided by considering their highly un­favorable prior probabilities. Others would require explicit sampling of the multiple minima.

#### 3.2.3. Lower resolution: peptide orientations with *CaBLAM* and Cβd outliers   

**Figure 8:**
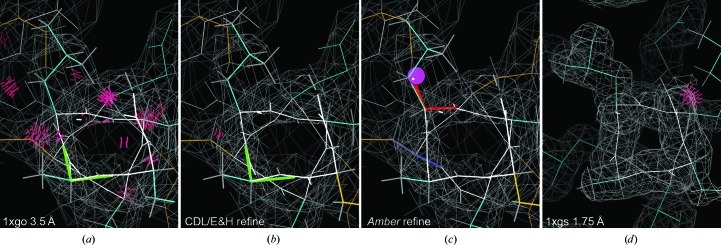
A C^β^ deviation in the *Amber* results at 3.5 Å resolution, but not for either the original or the CDL/E&H results. (*a*) Leu253 in PDB entry 1xgo on a quite distorted helix, with many clashes and a Ramachandran outlier; the Leu rotamer is incorrect, as shown by the structure with PDB code 1xgs at 1.75 Å resolution. (*b*) CDL/E&H refinement fixes the clashes, but not the rotamer or Ramachandran outliers or the helix distortion. (*c*) *Amber* refinement fixes the clashes and the Ramachandran outlier, flags the incorrect Leu rotamer with a Cβd outlier and moves the helix conformation closer to ideal. (*d*) Leu253 in PDB entry 1xgs at 1.75 Å resolution, with a clearly correct rotamer on an ideal helix and no outliers besides one clash.

At low resolution (2.5–4 Å), no waters or alternates are modeled. All other problems continue, but an additional set of common local misfittings occur because the broad electron density is compatible with significantly different models. PDB entry 1xgo at 3.5 Å resolution is an excellent case for testing in this range, because it was solved independently from the 1.75 Å resolution structure with PDB code 1xgs: the same molecule in a different space group. CDL/E&H refinement shows no Cβd outliers, but *Amber* refinement shows six. Comparison with PDB entry 1xgs shows that each of the Cβd residues has the side chain, the backbone or both in an incorrect local-minimum conformation uncorrectable by minimization refinement methods (Richardson & Richardson, 2018 ▸). For example, Fig. 8 ▸ shows Leu253 on a helix, with a Cβd from *Amber* (Fig. 8 ▸ *c*) and the different, correct PDB entry 1xgs Leu rotamer (Fig. 8 ▸ *d*). These Cβd outliers are thus a feature, not a bug, in *Amber*: they serve their designed validation function of flagging genuine fitting problems. However, the lack of Cβd outliers in the CDL/E&H refinement is also not a defect because the tight CDL/E&H geometry is on average quite useful at low resolution.

The 1xgo versus 1xgs comparison also illustrates many of the ways in which *Amber* refinement is superior at low resolution. In Fig. 8 ▸, *Amber* corrects a Ramachandran outlier in the helix and shows a helix backbone shape much closer to the ideal geometry of PDB entry 1xgs than either the deposited or the CDL/E&H versions.

**Figure 9:**
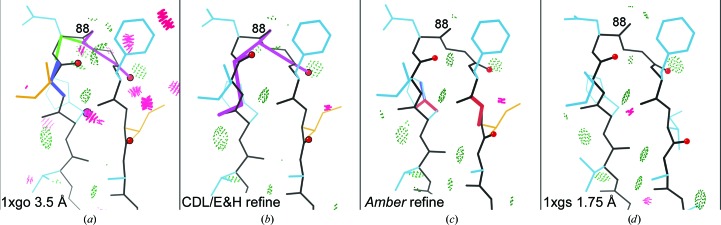
Two misoriented peptides in PDB entry 1xgo, flagged by Ramachandran and *CaBLAM* outliers (magenta outlines on the CO virtual dihedrals). (*a*) Residues 86–91 in the deposited structure with PDB code 1xgo. (*b)* CDL/E&H result, with unchanged conformation and outliers. (*c*) *Amber* result, with several peptide orientations changed by modest amounts (red spheres on CO), removing the backbone outliers and very closely matching the conformation for PDB entry 1xgs shown in (*d*).

Since the backbone CO direction cannot be seen at low resolution, the commonest local misfitting is a misoriented peptide (Richardson *et al.*, 2018 ▸). These can be flagged by the new *MolProbity* validation called *CaBLAM*, which tests whether adjacent CO directions are compatible with the local C^α^ backbone conformation (Williams, Headd *et al.*, 2018 ▸). Ten such cases were identified in PDB entry 1xgo for isolated single or double *CaBLAM* outliers surrounded by correct structure as judged in PDB entry 1xgs. In six of those ten cases neither CDL/E&H nor *Amber* refinement corrected the problem (His62, Thr70, Gly163, Gly193, Ala217 and Glu286; see Supplementary Fig. S2). In two cases CDL/E&H had fewer other outliers than *Amber* refinement, but did not actually reorient the CO (Gly193 and the Gly163 case shown in Supplementary Fig. S3). In three of the ten cases *Amber* performed a complete fix, while CDL/E&H did not provide any improvement (Asp88, Gly125 and Pro266). For example, in Fig. 9 ▸, residues 86–91 of PDB entry 1xgo (Fig. 9 ▸ *a*) have a *CaBLAM* outlier (magenta lines) uncorrected by CDL/E&H refinement (Fig. 9 ▸ *b*). However, *Amber* refinement (Fig. 9 ▸ *c*) manages to shift several CO orientations by modest amounts (red spheres), which is sufficient to fix the *CaBLAM* outliers and match the better backbone conformation of PDB entry 1xgs extremely closely (Fig. 9 ▸ *d*). The Gly125 example is shown in Supplementary Fig. S4. Finally, in one especially interesting case (Lys22) *Amber* turned the CO about halfway up to where it should be, while CDL/E&H made no improvement. The *Amber* model still has geometry outliers and further runs moved the CO most of the way up and removed those outliers, showing that *Amber* refinement had not yet fully converged in ten macrocycles (see the supporting information and Supplementary Fig. S5).

**Figure 10:**
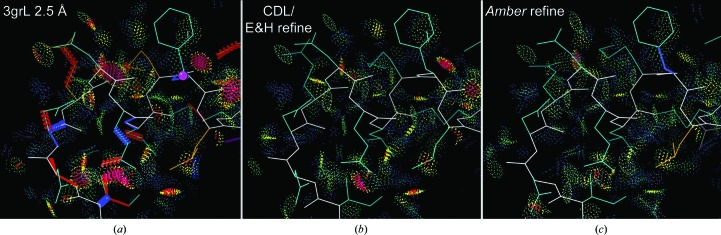
*Amber* refinement produces better hydrogen bonds and van der Waals contacts as well as removing somewhat more steric clashes. (*a*) The Asn182 helix-cap region in PDB entry 3g8L at 2.5 Å resolution, with numerous clashes and other outliers. (*b*) CDL/E&H refinement makes large improvements, removing most clashes and all other outliers. (*c*) *Amber* refinement does even better, removing all clashes and most small overlaps (yellow) and optimizing to produce more hydrogen bonds and favorable van der Waals contacts (green and blue dots).

**Figure 11:**
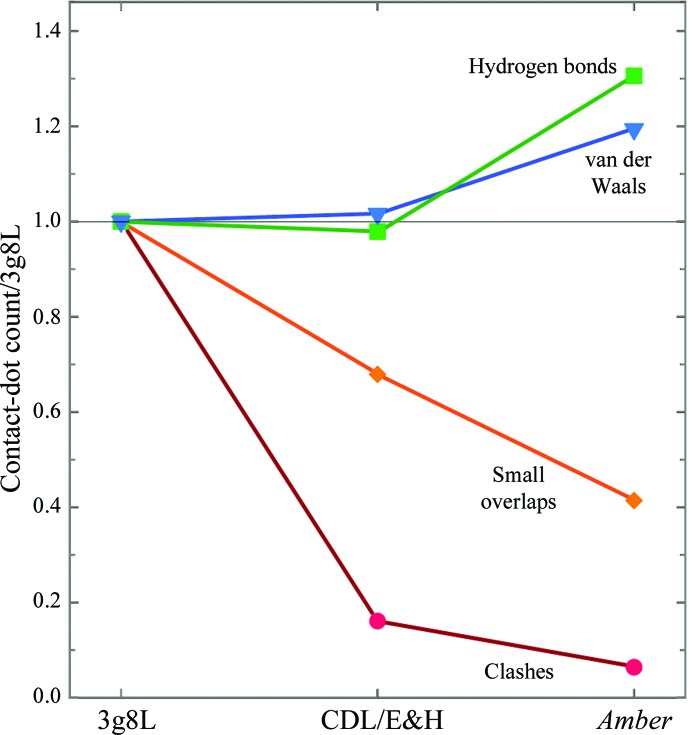
CDL/E&H versus *Amber* improvements in steric contacts for the helix-cap in PDB entry 3g8L, quantified by all-atom contact dot or spike counts measured in *Mage* (Richardson & Richardson, 2001 ▸), normalized relative to the counts in the deposited structure with PDB code 3g8L. *Amber* changes farthest, in the right direction, for all four contact types.

*Amber* refinement is especially good at optimizing hydrogen-aware all-atom sterics, as calculated by *Probe* (Word, Lovell, LaBean *et al.*, 1999 ▸) with H atoms added and optimized by *Reduce* (Word, Lovell, Richardson *et al.*, 1999 ▸). This is illustrated in Fig. 10 ▸ for PDB entry 3g8L at 2.5 Å resolution. The deposited structure of the Asn182 helix N-cap region, which has many outliers of all kinds (Fig. 10 ▸ *a*), is improved a great deal by CDL/E&H refinement (Fig. 10 ▸ *b*). However, the *Amber* refinement (Fig. 10 ▸ *c*) is noticeably better, with more hydrogen bonds and better van der Waals contacts, as well as fewer clashes. These improvements are plotted quantitatively in Fig. 11 ▸, as measured by a decrease in un­favorable clash spikes (red) and small overlaps (orange), with an increase in favorable hydrogen bonds (green) and van der Waals contacts (blue).

## 4. Discussion   

The idea of including molecular-mechanics force fields into crystallographic refinements is not a new one, with precedents dating back to early work by Jack & Levitt (1978 ▸) and the *X-PLOR* program (Brünger & Karplus, 1991 ▸) developed in the 1980s. The notion that a force field could (at least in principle) encode ‘prior knowledge’ about protein structure continues to have a strong appeal, and efforts to replace conventional ‘geometric restraints’, which are very local and uncorrelated, with a more global assessment of structural quality have been explored repeatedly (see, for example, Moulinier *et al.*, 2003 ▸; Schnieders *et al.*, 2009 ▸). Distinguishing features of the current implementation include the automatic preparation of force fields for many types of biomolecules, ligands and solvent components as well as close integration with *Phenix*, a mature and widely used platform for refinement. This has enabled parallel refinements on more than 22 000 protein entries in the PDB and allows crystallographers to test these ideas on their own systems by simply adding flags to an existing *phenix.refine* command line or adding the same information via the *Phenix* GUI. Indeed, we expect most users to ‘turn on’ *Amber* restraints after having carried out a more conventional refinement to judge for themselves the significance and correctness of the structural differences that arise. As noted in Section 3.2, an *Amber* refinement will often flag residues that need manual refitting in ways complementary to the cues provided by more conventional refinement.

The results presented here show that structures with improved local quality (as monitored by *MolProbity* criteria and hydrogen-bond analysis) can be obtained by simple energy minimization, with minimal degradation in the agreement with experimental structure factors and with no changes to a current-generation protein force field. Nevertheless, one should keep in mind that the *Amber*-refined structures obtained here are not very different from those found using more conventional refinement. Both methods require that most local misfittings be corrected in advance. The hope is that either sampling of explicit alternatives or else optimization using more aggressive conformational search, such as with simulated annealing or torsion-angle dynamics, may find the correct low-energy structures with good agreement with experimental data.

It is likely that further exploration of relative weights between ‘X-ray’ and ‘energy’ terms (beyond the existing and heuristic weight-optimization procedure employed here), and even within the energy terms, will become important. In principle, maximizing the joint probability arising from ‘prior knowledge’ [using a Boltzmann distribution, exp(−*E*_AmberFF_/*k*_B_ *T*), for some effective temperature] and a maximum-likelihood target function (based on a given model and the observed data) is an attractive approach that effectively establishes an appropriate relative weighting. More study will be needed to see how well this works in practice, especially in light of the inevitable limitations of current force fields.

The integration of the Amber force field into the *Phenix* software for crystallography also paves the way for the development of more sophisticated applications. The force field can accommodate alternate conformers by using the locally enhanced sampling (LES) approach (Roitberg & Elber, 1991 ▸; Simmerling *et al.*, 1998 ▸); a few examples are discussed here, whilst details will be presented elsewhere. Ensemble refinement (Burnley *et al.*, 2012 ▸) could now be performed using a full molecular-dynamics force field, thus avoiding poor-quality individual models in the ensemble. Similarly, simulated annealing could now be performed with an improved physics-based potential. Extension of the ideas presented to real-space refinement within *Phenix* is under way, opening a path to new applications to cryo-EM and low-resolution X-ray structures. These developments would all contribute significantly to the future of macromolecular crystallography, reinforcing the transition from a single static structure-dominated view of crystals to one in which dynamics and structural ensembles play a central important role in describing molecular function (Furnham *et al.*, 2006 ▸; van den Bedem & Fraser, 2015 ▸; Wall *et al.*, 2014 ▸).

## 5. Conclusions   

We have presented refinement results obtained by integrating *Phenix* with the *Amber* software package for molecular dynamics. Our refinements of over 22 000 crystal structures show that refinement using the Amber all-atom molecular-mechanics force field outperforms CDL/E&H restraint refinement in many respects. An overwhelming majority of *Amber*-refined models display notably improved model quality. The improvement is seen across most indicators of model quality, including clashes between atoms, side-chain rotamers and peptide-backbone torsion angles. In particular, *Phenix*–*Amber* consistently outperforms standard *Phenix* refinement in clashscore, number of hydrogen bonds per 1000 atoms and *MolProbity* score. It also consistently outperforms standard refinement for Ramachandran and rotamer statistics at low resolutions and obtains approximately equal results at high (better than 2.0 Å) resolutions. *Amber* does run somewhat more slowly (generally taking 20–40% longer) and may take more cycles for a particular local conformation to converge completely if it is making a large local change (see the caption to Supplementary Fig. S5). It should be noted that standard refinement consistently outperforms *Phenix*–*Amber* in eliminating C^β^ deviation and other covalent-geometry outliers across all resolutions, but in most cases the *Amber* outliers serve to flag a real problem in the model.

As the quality of experimental data decreases with resolution, the improvement in model quality obtained by using *Amber*, as opposed to CDL/E&H restraints, increases. This improvement is especially striking in the case of clashscores, which appear to be nearly independent of experimental data resolution for *Amber* refinements. Additional improvement is seen in the modeling of electrostatic interactions, hydrogen bonds and van der Waals contacts, which are currently ignored by conventional restraints. Improving lower resolution structures is very important, since they include a large fraction of the most exciting and biologically important current structures such as the protein/nucleic acid complexes of large, dynamic molecular machines.

No minimization refinement method, including CDL/E&H and *Amber*, can in general correct local misfittings that were modeled in an incorrect local minimum conformation, especially at relatively high resolutions. At lower resolutions, where the barriers are softer, *Amber* can sometimes manage such a change, while CDL/E&H still does not. It is, therefore, important and highly recommended that validation flags be consulted for the initial model and as many of the worst cases be fixed as feasible before starting the cycles of automated refinement with either target.

## 6. Software distribution   

*Amber* was implemented in *phenix.refine* and is available in v.1.16-3549 of *Phenix* and later. Instructions for using the *phenix.refine* *Amber* implementation are available in the version-specific documentation available with the distribution. The *Amber* codes are included in the *Phenix* distribution under the terms of the GNU lesser general public license (LGPL).

## 7. Related literature   

The following references are cited in the supporting information for this article: Jorgensen *et al.* (1983 ▸), Joung & Cheatham (2009 ▸), Tahirov *et al.* (1998 ▸) and Wang *et al.* (2004 ▸, 2006 ▸).

## Supplementary Material

Supplementary information and figures. DOI: 10.1107/S2059798319015134/lp5044sup1.pdf
