# Supplementary material for: Improved chemistry restraints for crystallographic refinement by integrating the Amber force field into Phenix
Source: Acta Crystallogr D Struct Biol. 2020 Jan 1;76(Pt 1):51–62. doi: 10.1107/S2059798319015134 (PMC6939439; doi:10.1107/S2059798319015134)
Supplement: Supplementary file 1 [file d-76-00051-sup1.pdf]

STRUCTURAL
BIOLOGY

Volume 75 (2019)

Supporting information for article:

Improved chemistry restraints for crystallographic refinement by integrating the Amber force field into *Phenix*

Nigel W. Moriarty, Pawel A. Janowski, Jason M. Swails, Hai Nguyen, Jane S. Richardson, David A. Case and Paul D. Adams

S1. Preparation of structures for Phenix-Amber refinement.

The *AmberPrep* program prepares the files needed for the subsequent refinement step. For components (typically ligands) that are not standard amino acids, nucleotides, solvent or monatomic ions, the eLBOW routines (Moriarty *et al.,* 2009) are used to add hydrogen atoms and determine the most likely protonation and tautomeric states. These three-dimensional structures are then used in the standard way in Amber's antechamber tool (Wang *et al.,* 2006) to assign charges and atom types using version 2.11 of the general Amber force field (GAFF) (Wang *et al.,* 2004). Proteins are modeled using the ff14SB force field (Maier *et al.,* 2015), water and related ions with the TIP3P model and associated parameters for ions (Jorgensen *et al.,* 1983; Joung & Cheatham, 2009).

This procedure will fail for ligands containing metal ions (since the GAFF force field currently only deals with organic moieties), and also for ligands that have covalent connections to the protein. For each of these cases, users familiar with the Amber software can build the needed component libraries using other Amber-based tools. Because such efforts are not yet fully automated, structures with metal-containing ligands or covalent connections were left out of the current caclulations.
After these component libraries are prepared, the coordinates of the system are expanded to a full unit cell, and Amber's *tleap* program is used to construct topology and coordinate files in Amber format. Disulfide bonds and gaps in the sequence are identified and properly processed. A model file in PDB format for the asymmetric unit (for use by Phenix) is also created that contains any added hydrogen atoms or missing atoms; any atomic displacement parameters (ADPs) from the input PDB file are copied to this file; hydrogen atoms are assigned isotropic B-factors that match the heavy atoms to which they are bonded. For the main statistical analysis, only the most populated alternate conformer was selected, and assigned unit occupancy. As discussed in the text, for a selected set of structures, we also used an option in the code to include all alternate conformers present in the input PDB file.

During refinement, *phenix.refine* sees only a single asymmetric unit, as usual. At each step, when Amber restraints are required, these coordinates are expanded to a full unit cell, the Amber force field is called to compute energies and gradients and the gradients for principal asymmetric unit are passed back to *phenix.refine* in place of conventional geometric restraints.

S2. Refinement parameters

Parameters used in both sets of refinements.

```
c_beta_restraints=False
discard_psi_phi=False
strategy=*individual_sites individual_sites_real_space rigid_body
   *individual_adp group_adp tls *occupancies group_anomalous
flip_symmetric_amino_acids=True
```

```
refinement.target_weights.optimize_xyz_weight=True
refinement.main.number_of_macro_cycles=10
```

S3. Full-dataset comparisons

Bond and angle rmsd comparisons (see figure S1) show that the bond rmsd values are numerically different but are smaller than the average sigma of 0.02Å (2pm) applied to protein bond restraints. Furthermore, the Amber angle rmsd values are approximately 2° across all resolutions – also lower than the average of ~3° applied to protein angle restraints. The increased CDL/E&H rmsd values at high resolution may be result of the looser rmsd limit used for better than 2Å for the weight optimisation process. Comparing the means of the CDL/E&H and Amber rmsd values is not valid as force fields use more complex energetics rather than harmonic targets to ideal values.

S4. Response to Bad Peptide Orientations

S4.1. Background

The low-resolution analysis of Cβ deviations in the main text made use of comparing the 1xgo structure at 3.5Å (Tahirov 1998) versus 1xgs at 1.75Å from the same paper. All six Cβ deviations in the Amber results versus none from CDL/E&H were compared, finding that in each case that Cβd was flagging an underlying problem: either a misfit side chain or an incompatibility between backbone and side chain.

For the issue of bad peptide orientations, however, only one example was illustrated (Figure 9). These problems are common at resolutions worse than 2.5Å, because the backbone CO direction is no longer seen (Richardson *et al*., 2018). Misoriented peptides can be diagnosed by CaBLAM (Williams 2018). CaBLAM uses virtual dihedral angles of successive Cαs and of successive COs to test whether the orientations of successive CO groups are compatible with the surrounding Cα trace. It flags outliers graphically in magenta on the CO-CO virtual dihedral. Since typically there is an energy barrier between widely different peptide orientations, the presumption is that refinement cannot easily correct these cases. However, that presumption needs to be tested.

S4.2. Most are not correctable by refinement

Ten cases were identified in 1xgo, for isolated single or double CaBLAM outliers (usually with other outliers also), surrounded by correct structure as judged in the same molecule at 1.75Å resolution (1xgs). For 6 of those 10 cases, neither CDL/E&H nor Amber refinement corrected the problem (His62, Thr70, Gly163, Gly193, Ala217, Glu286).

For example, figure S2 shows stereo images of the Glu286-Lys287 hairpin-loop case, where the CaBLAM outlier in 1xgo is accompanied by clashes, Ramachandran and rotamer outliers. Both CDL/E&H and Amber conformations are essentially identical to the original 1xgo, with no peptide

improvement. They both remove all the clashes (clusters of hotpink spikes) and remove one of the six side chain outliers (gold) but not into the correct rotamer. In contrast, the high-resolution 1xgs, with very clear electron density (bottom panel), shows the Lys Cα and the two peptide carbonyl oxygens (red balls) differently placed by large distances and dihedral angles, forming a well H-bonded β-hairpin with no outliers of any kind.

S4.3. Other Outliers Often Better

In two cases the CDL/E&H results had fewer other outliers than Amber, although it did not actually reorient the peptide CO (Gly163, Gly193). The Gly163 case is shown in stereo in figure S3, for an S-shaped loop between non-adjacent β-strands, with two CaBLAM flags (magenta) and many other outliers. Both refinements remove the clashes, one of the rotamer outliers and one of the Ramachandran outliers (green). The CDL/E&H results in addition removed one of the CaBLAM outliers and the Cα-geometry outlier (red). However, neither refinement could manage the large rotation needed to correct the 163-164 peptide orientation, as judged by the more convincing conformation of the high-resolution 1xgs at bottom.

S4.4. Amber Sometimes Corrects Well

In three cases Amber managed a complete fix, while in contrast CDL/E&H did not improve (Asp88, Gly125, Pro266). The Asp88-Gly89 tight turn example is shown in Figure 9 of the main text.

Here in figure S4, the Gly125 loop example in a helix-helix connection is shown in stereo, to allow clear visualization of the CO orientation changes. 1xgo residues 121-126 (figure S3a) have two CaBLAM outliers (magenta dihedral lines) unchanged by CDL/E&H refinement (panel b). However, Amber refinement (panel c) manages to shift several CO orientations by up to 80° (red balls), enough to fix the CaBLAM outliers and to match extremely closely the better backbone conformation of 1xgs (panel d).

S4.5. A Partial Correction, Unconverged

Finally, in one especially interesting case (Lys22, in Figure S5a for 1xgo) Amber turned the CO (red circles) about halfway up to where it should be (panels b vs c), while CDL/E&H made no improvement to the peptide. The Amber model eliminated the Ramachandran and one of the CaBLAM outliers, but still had geometry outliers (a bond angle and a Cβ deviation). It seemed likely that Amber refinement had not fully converged and might move the CO all the way if run longer.

A 30-cycle Amber run had earlier been done for 1xgo, without any major changes noticed beyond the 10-cycle. From that endpoint, two further runs were done, first of 30 cycles ("Amber60"), then a further 10 cycles ("Amber70").

Figure S5d shows the fan of CO positions for all 7 of the deposits and refinements, progressively rotating counterclockwise from 1xgo to 1xgs. Indeed, both Amber60 and Amber70 successfully rotated the Lys22 peptide almost all the way to the good helical position seen in the high-resolution 1xsg (panel e), eliminating both the CaBLAM outlier and the intermediate-stage bond-angle outliers, presumably having crossed an energy barrier in the process.

One other CaBLAM-outlier peptide was corrected in Amber70 as well (Thr71). But for the Ala217 outlier, the wrong peptide was rotated, seduced by H-bonding to an Arg side chain in the wrong position.

In these long refinements, both R-factors and match to electron density suffer somewhat. In the cases examined, this often seems due to incorrect side chain rotamers (almost never correctable by refinement) pushing an otherwise-good backbone conformation a bit out of density (translated upward, for 1xgo Lys22). Future work will try to guide early correction of as many problems as feasible, for the faster and more successful refinement afterward that we now know is possible.

S4.6. Discussion

In summary, it is indeed true that refinement cannot usually correct a peptide orientation that is off by a large amount. The very tight geometry restraints in the CDL/E&H system presumably raise the barriers to peptide rotation. Amber is rather better at that, and about 1/3 of the time managed a good correction, although convergence can be very slow for such large changes. We feel it is crucial to try correcting problems such as flipped peptides in the initial model before refining it, however, crosstalk between backbone and side chains further complicates that process. However, we are enthusiastic about use of the Amber target to realistically improve conformation and especially sterics, once the model is mostly in the right local minima.

S5. Boxplots of MolProbity results

Boxplots of the MolProbity results are given in figure S6 and S7. The latter has the difference between the CDL/E&H refinements and the Amber refinements. The largely (approximately 70% of all comparison) positive values show that Amber lowers the MolProbity score in the majority of cases.

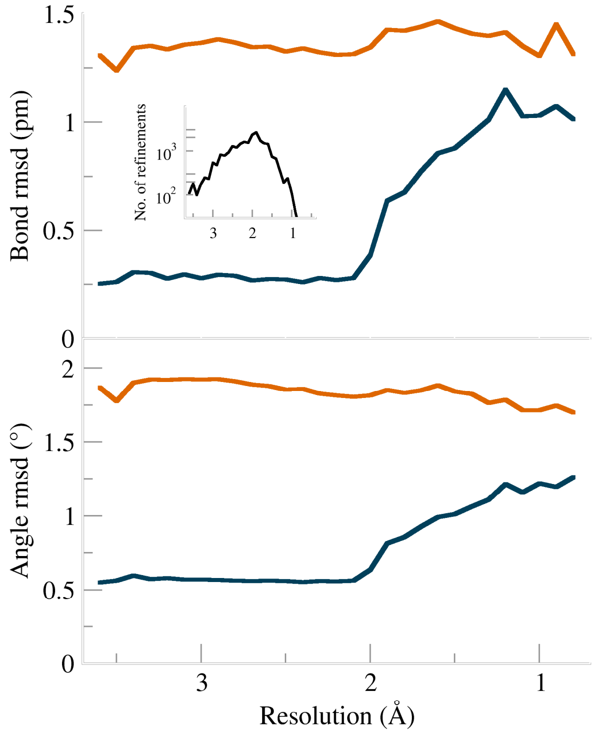

Figure S1 Bond and angle rmsd values for CDL/E&H (dark blue) and Amber (burnt orange) plotted against resolution.

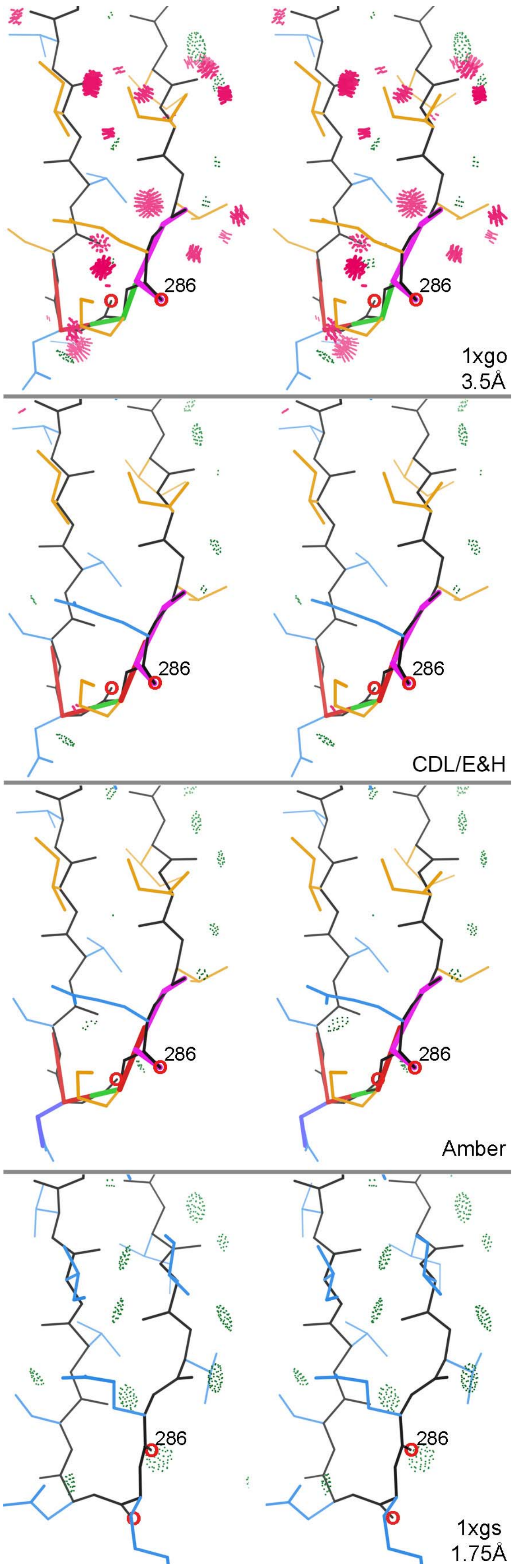
286
286
1xgo
3.5Å
286
286
CDL/E&H
286
286
Amber
286
286
1xgs
1.75Å

Figure S2 Stereo images of uncorrected CaBLAM problems for the beta-hairpin loop at Glu 286 - Lys 287 in 1xgo at 3.5Å resolution. a) As deposited, with outliers for CaBLAM (magenta lines on the CO dihedral), CaBLAM Cα-geometry (red lines on Cα trace), Ramachandran (green lines along backbone), rotamer (gold sidechains), and all-atom clash (clusters of hot-pink spikes) evaluations. b) As refined by Phenix CDL/E&H and c) as refined by Phenix Amber, both of which remove the clashes but do not correct the underlying conformation. d) In the 1xgs structure at 1.75Å resolution, showing a classic, outlier-free beta hairpin conformation with good backbone H-bonding and substantial corrections in peptide orientation and sidechain placement. The 286 and 287 peptide oxygens that move most are circled in red.

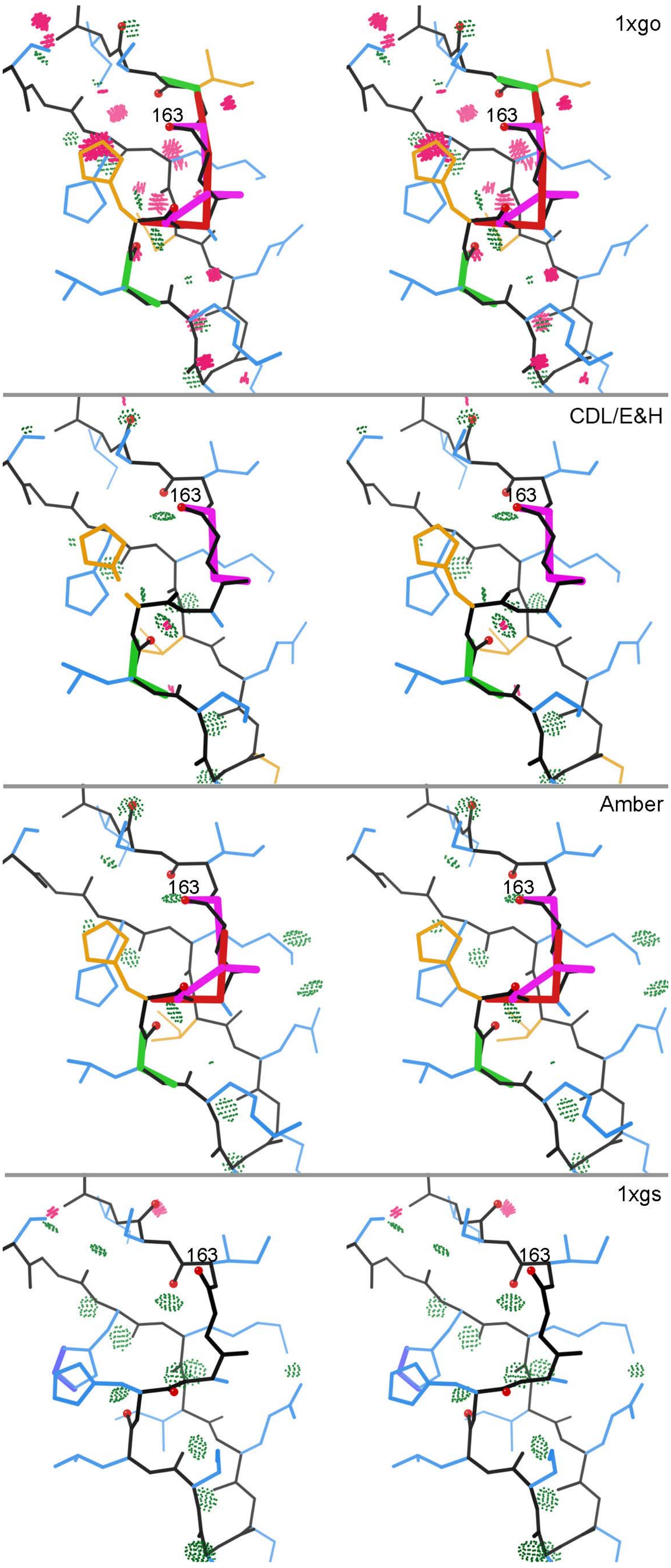
1xgo
163
163
CDL/E&H
163
163
Amber
163
163
1xgs
163
163

Figure S3 Partial correction of an S-shaped loop at 159-164 in 1xgo. a) As deposited, with many types of outliers. b) CDL/E&H corrects all but two backbone outliers. c) Amber corrects all clashes but few other outliers, and neither refinement changes the poor underlying conformation. d) The 1xgs structure achieves an outlier-free, well H-bonded conformation by shifting 4 peptide orientations (red ball on carbonyl O atoms), especially at Gly 163.

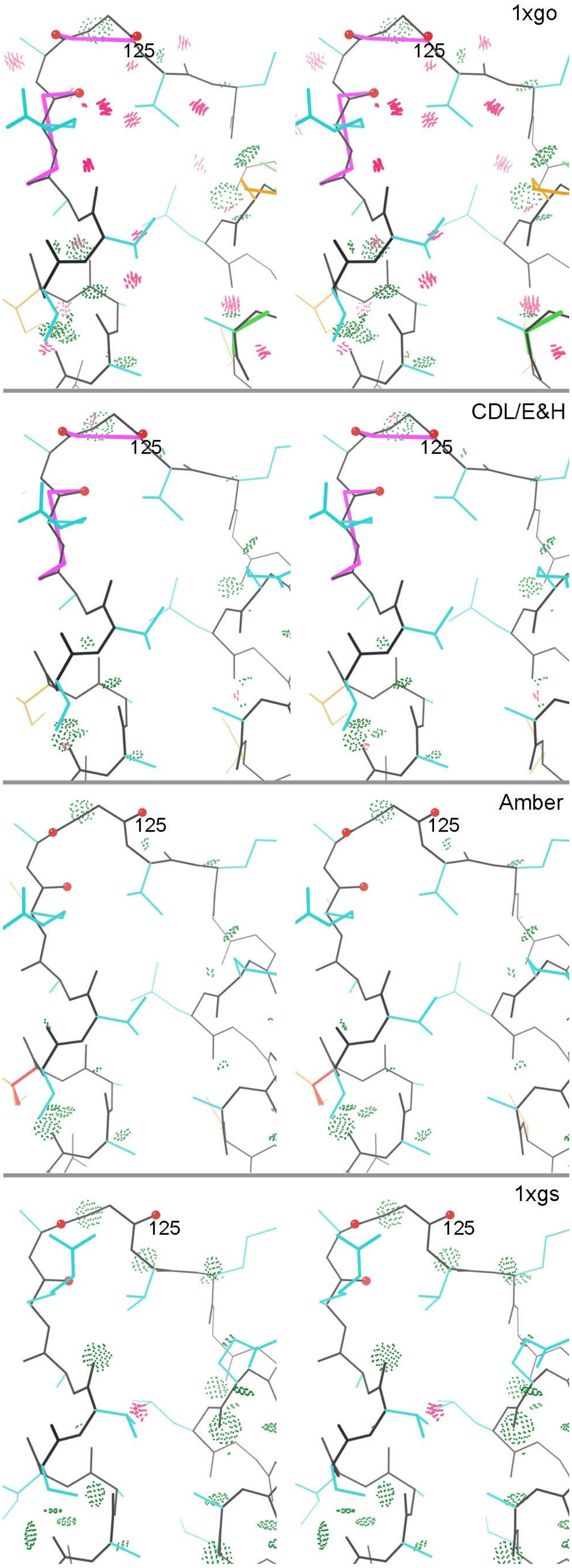
1xgo
125
125
CDL/E&H
125
125
Amber
125
125
1xgs
125
125

Figure S4 Successful Amber CaBLAM corrections in the helix-helix loop at 1xgo 121-126. a) As deposited, with clashes and two CaBLAM outliers. a) CDL/E&H corrects the clashes but not the backbone conformation. b) Amber reorients 3 successive peptides (red balls on peptide Os) by up to 80°, removing both CaBLAM outliers and matching extremely closely the conformation seen at high resolution in panel d.

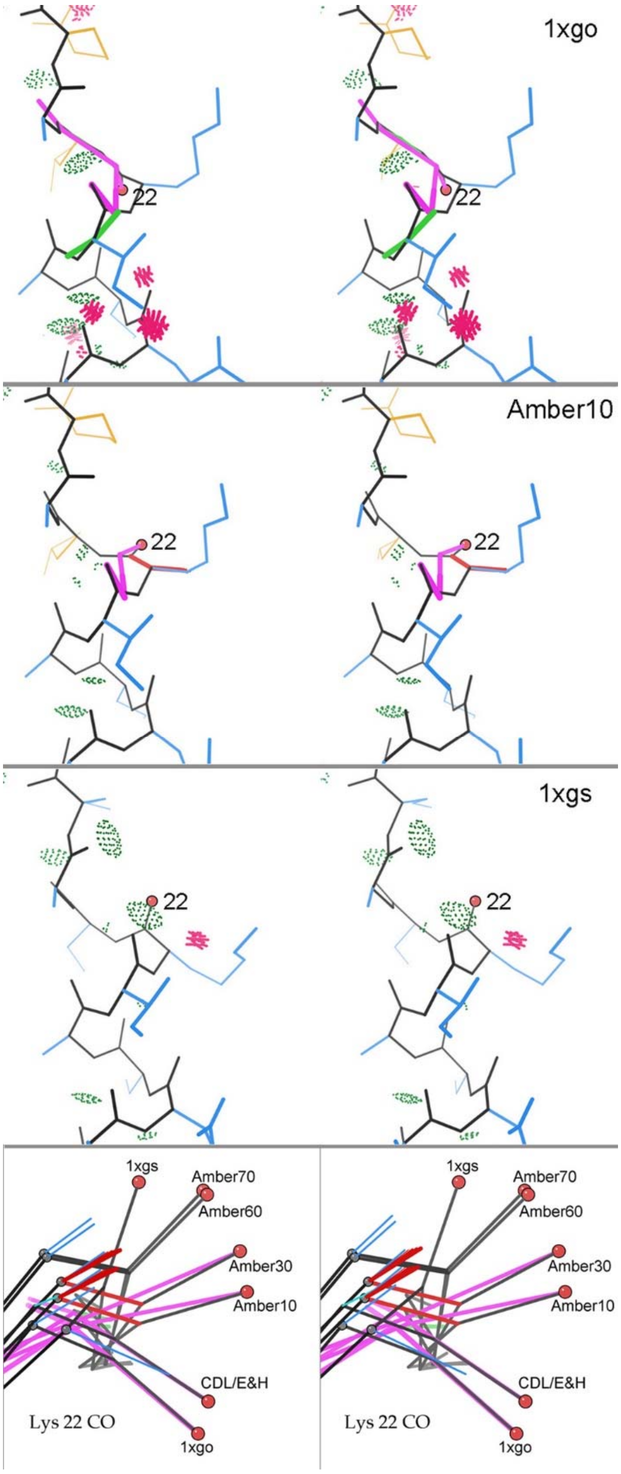

Figure S5 Gradual correction of the helix C-cap at 1xgo Lys 22. a) As deposited, with double CaBLAM outliers, clashes, and Ramachandran outlier. CDL/E&H refinement fixes clashes but leaves conformation unchanged. b) Amber refinement moves the crucial Lys 22 CO partway up toward α-helical orientation, relieving one of the CaBLAM outiers. c) Helical, outlier-free conformation of the C-cap region in 1xgs at high resolution. d) Superposition in side view, showing all Lys 22 CO orientations between 1xgo outlier and 1xgs α-helical: longer Amber refinement progressively corrects the orientation, converging close to the 1xgs orientation although with a translational shift we believe is an effect of incorrect sidechain rotamers.

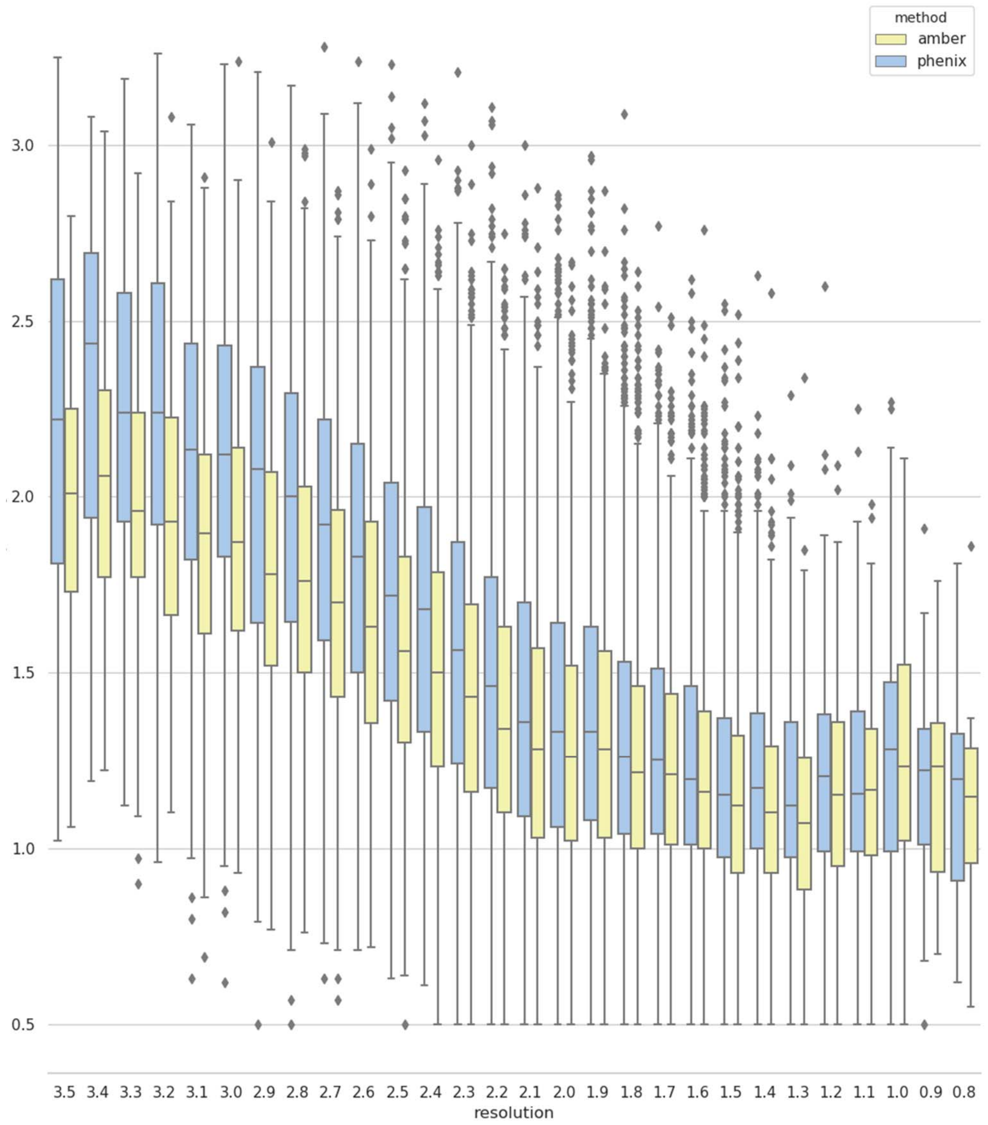

Figure S6 Molprobity scores binned into 0.1Å bins and plotted as boxplots. The box portion of the plot indicates the three quartile values while the whiskers cover the range of the values. Dots are outliers determined via a method of inter-quartile range.

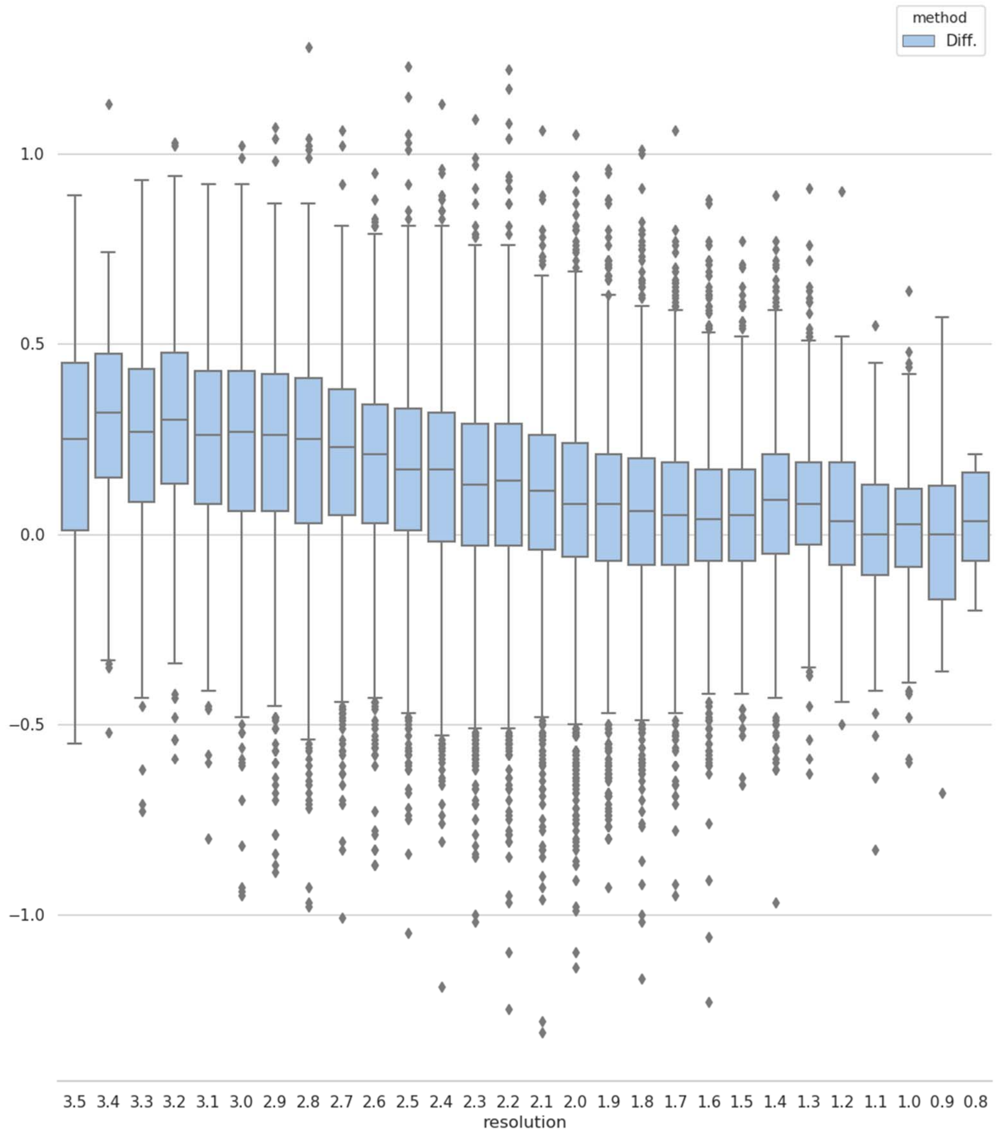

Figure S7 Differences in MolProbity scores between the Phenix and Phenix-Amber values (see figure S6) binned into 0.1Å bins and plotted as boxplots. Positive values indicate that Phenix-Amber refinements improved (decreased) the MolProbity score. The box portion of the plot indicates the three quartile values while the whiskers cover the range of the values. Dots are outliers determined via a method of inter-quartile range.

Jorgensen, W. L., Chandrasekhar, J., Madura, J. D., Impey, R. W. & Klein, M. L. (1983). *J. Chem. Phys.* **79**, 926–935.

Joung, I. S. & Cheatham, T. E. (2009). *J. Phys. Chem. B*. **113**, 13279–13290.

Maier, J. A., Martinez, C., Kasavajhala, K., Wickstrom, L., Hauser, K. E. & Simmerling, C. (2015). *J. Chem. Theory Comput.* **11**, 3696–3713.

Moriarty, N. W., Grosse-Kunstleve, R. W. & Adams, P. D. (2009). Acta Crystallogr. Sect. -Biol. Crystallogr. 65, 1074–1080.

Richardson J, Richardson D (2018) Cβ deviations and other aspects in Amber versus CDL refinements, *Comp. Cryst. Newsletter* **9**: 21-24

Richardson JS, Williams CJ, Videau LL, Chen VB, Richardson DC (2018) "Assessment of detailed conformations suggests strategies for improving cryoEM models: helix at lower resolution, ensembles, pre-refinement fixups, and validation at a multi-residue length scale", *J Struct. Biol.* **204**: 301-312

Tahirov TH, Oki H, Tsukihara T, Ogasahara K, Yutani K, Ogata K, Izu Y, Tsunasawa S, Kato I (1998) Crystal structure of methionine aminopeptidase from hyperthermophile *Pyrococcus furiosus, J. Mol. Biol.* **284**: 101-124Wang, J., Wang, W., Kollman, P. A. & Case, D. A. (2006). *J. Mol. Graph. Model.* **25**, 247–260.

Wang, J., Wolf, R. M., Caldwell, J. W., Kollman, P. A. & Case, D. A. (2004). *J. Comput. Chem.* **25**, 1157–1174.

Williams CJ, Hintze BJ, Headd JJ, Moriarty NW, Chen VB, Jain S, Prisant MG Lewis SM, Videau LL, Keedy DA, Deis LN, Arendall WB III, Verma V, Snoeyink JS, Adams PD, Lovell SC, Richardson JS, Richardson DC (2018) MolProbity: More and better reference data for improved all-atom structure validation, *Protein Sci.* **27**: 293–315
